# Historical Shifts in Brazilian *P. falciparum* Population Structure and Drug Resistance Alleles

**DOI:** 10.1371/journal.pone.0058984

**Published:** 2013-03-15

**Authors:** Sean M. Griffing, Giselle M. Rachid Viana, Tonya Mixson-Hayden, Sankar Sridaran, Mohammad Tauqeer Alam, Alexandre Macedo de Oliveira, John W. Barnwell, Ananias A. Escalante, Marinete Marins Povoa, Venkatachalam Udhayakumar

**Affiliations:** 1 Malaria Branch, Division of Parasitic Diseases and Malaria, Center for Global Health, Centers for Disease Control and Prevention, Atlanta, Georgia, United States of America; 2 Atlanta Research and Education Foundation, Decatur, Georgia, United States of America; 3 Laboratório de Pesquisas Básicas em Malária, Seção de Parasitologia, Instituto Evandro Chagas, Brazil; 4 Association of Public Health Laboratories, Silver Spring, Maryland, United States of America; 5 School of Life Sciences, Arizona State University, Tempe, Arizona, United States of America; Instituto de Higiene e Medicina Tropical, Portugal

## Abstract

Previous work suggests that Brazilian *Plasmodium falciparum* has limited genetic diversity and a history of bottlenecks, multiple reintroductions due to human migration, and clonal expansions. We hypothesized that Brazilian *P. falciparum* would exhibit clonal structure. We examined isolates collected across two decades from Amapá, Rondônia, and Pará state (n = 190). By examining more microsatellites markers on more chromosomes than previous studies, we hoped to define the extent of low diversity, linkage disequilibrium, bottlenecks, population structure, and parasite migration within Brazil. We used retrospective genotyping of samples from the 1980s and 1990s to explore the population genetics of SP resistant *dhfr* and *dhps* alleles. We tested an existing hypothesis that the triple mutant *dhfr* mutations 50R/51I/108N and 51I/108N/164L developed in southern Amazon from a single origin of common or similar parasites. We found that Brazilian *P. falciparum* had limited genetic diversity and isolation by distance was rejected, which suggests it underwent bottlenecks followed by migration between sites. Unlike Peru, there appeared to be gene flow across the Brazilian Amazon basin. We were unable to divide parasite populations by clonal lineages and pairwise FST were common. Most parasite diversity was found within sites in the Brazilian Amazon, according to AMOVA. Our results challenge the hypothesis that triple mutant alleles arose from a single lineage in the Southern Amazon. SP resistance, at both the double and triple mutant stages, developed twice and potentially in different regions of the Brazilian Amazon. We would have required samples from before the 1980s to describe how SP resistance spread across the basin or describe the complex internal migration of Brazilian parasites after the colonization efforts of past decades. The Brazilian Amazon basin may have sufficient internal migration for drug resistance reported in any particular region to rapidly spread to other parts of basin under similar drug pressure.

## Introduction

Malaria has been reported in Brazil since the 1500s when *Plasmodium falciparum* spread along the coast with the sugarcane industry. Migration patterns driven by later industry (gold panning, diamond mining, rubber tapping, agriculture, cattle ranching, etc) and colonization propagated malaria epidemics and the principal vector, *Anopheles darlingi* into the interior [1], [2], [3]. By the early 1940s, there were six million malaria cases per year in Brazil, which was then a seventh of the population [4]. After the introduction of DDT and chloroquine treatment, there were only 28,557 *P. falciparum* cases in 1970 among 92.3 million people and 73% of cases were reported in the Amazon region [4], [5], [6], [7], [8].

During the 1970s, immunological naïve people from the south and elsewhere migrated to the gold mining areas in the Amazon, particularly Rondônia [9], [10], [11]. The construction of roads, hydroelectric plants, livestock and agricultural projects, mines, and other infrastructural project also led to an increase in malaria transmission [6]. *Plasmodium* migrated between the various malarious regions in multiple directions [12]. Malaria cases tripled between 1970 and 1980 due to the removal of DDT, reduction of control efforts and integration with other public health services, deforestation of the Amazon, and drug resistance [6], [8], [13], [14], [15], [16]. By 1990, 99% of malaria in Brazil occurred in states around the Amazon basin: Acre, Amapá, Amazonas, Maranhão, Mato Grosso, Pará, Rondônia, Roraima and Tocantins [6].

Sulfadoxine pyrimethamine (SP) resistance developed relatively quickly. Pyrimathemine in combination with various sulphonamides was first used in Brazil during the early 1960s and low level pyrimethamine resistance was then reported at sites throughout the country [17], [18]. Resistance to SP was first reported in Goiás in 1972 and six years later in Maranhão [17], [19]. In 1982, RIII SP resistance was reported along the road between Manaus, Amazonas, and Porto Velho, Rondônia [19]. By the end of the 1980s, 90% of parasites were SP resistant.

Existing work on Brazilian *P. falciparum* suggests that there is limited genetic diversity as seen in other South American countries [20], [21], [22], [23], [24], [25], [26]. When combined with the history of Brazilian malaria, it appears that Brazilian *P. falciparum* may have gone through bottlenecks, multiple reintroductions due to human migration, and potentially clonal expansions. We previously showed that Peruvian *P. falciparum* populations were highly clonal [27] and here we used the same tools to examine Brazilian *P. falciparum*. Our expectation was that Brazilian parasites might have similar population structure. We examined isolates collected across Brazil through two decades from Amapá, Rondônia, and Pará state. By examining more microsatellites markers on more chromosomes than previous studies, we hoped to define the extent of low diversity, linkage disequilibrium, bottlenecks, population structure, and parasite migration within Brazil. There is little information regarding the prevalence of SP resistant *dhfr* and *dhps* alleles over time in Brazil and we used retrospective genotyping of samples from the 1980s and 1990s to explore their population genetics. The availability of historical samples from different sites in Brazil also provided the chance to test an existing hypothesis that the triple mutant *dhfr* mutations 50R/51I/108N and 51I/108N/164L developed in southern Amazon from a single origin of common or similar parasites [28].

## Methods

### Ethics Statement

Samples used in this study were collected previously for malaria surveillance of drug resistance with the approval of Brazilian institutional ethical review boards (Instituto Evandro Chagas, Belem and Brazilian Ministry of Health) and approved by the U.S. Centers for Disease Control and Prevention for retrospective investigation. Participants provided written consent.

### Study sites and *P. falciparum* clinical isolates

**Figure 1:**
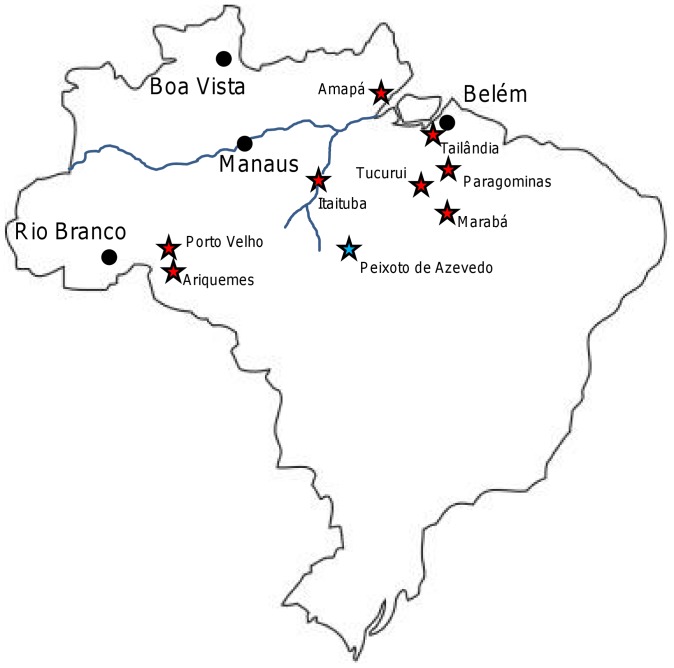
All Sample Sites. The map shows all sites examined during this study. They are marked with red stars. The blue line represents the Amazon River and a major tributary.

We examined 190 Brazilian samples collected during the 1980s and 1990s in Brazil in Amapá, Pará, and Rondônia (Table 1; Figure 1). Amapá is a coastal state in the northeast known for gold mining. Pará is a coastal state just to the south which includes the terminus of the Amazon River, a major port city (Belém), and various hydroelectric projects. Rondônia, in the west of the country, is known for rubber tapping, gold mining, and 20^th^ century subsistence farmers.

**Table 1:** Samples used in this study.

Brazilian State	Location	Year	Number of Samples
Para	Itaituba	1983	2
-	-	1984	1
-	-	1985	4
-	-	1986	2
-	-	1999	23
-	Marabá	1984	2
-	-	1986	1
-	-	1998	22
-	-	1999	18
-	Tailândia	1998	30
-	Tucuruí	1983	1
-	-	1984	2
-	Paragominas	1983	2
-	-	1984	2
-	-	1985	1
Amapá	-	1983	1
-	-	1984	1
-	-	1985	9
-	-	1986	1
Rondônia	Ariquemes	1984	1
-	-	1988	2
-	-	1996	2
-	Porto Velho	1996	19
-	-	1998	27
-	-	1999	13

### DNA isolation, PCR amplification and genotyping of *dhfr* and *dhps*

DNA was isolated from filter paper blood spots [29], [30], [31] or whole blood [29] using the QIAamp DNA blood mini kit (QIAGEN, Valencia, CA). Isolates were sequenced for point mutations in *dhfr*and *dhps* using methods previously described [27], [29], [32], [33], [34].

### Microsatellite typing

Whole genome amplified DNA (Qiagen's REPLI-g Whole Genome Amplification Kit, Valencia, CA) was used for microsatellite characterization. Samples were assayed for; 12 microsatellite loci spanning 700 kb around *dhfr* on chromosome 4; and 16 microsatellite loci spanning 406.3 kb around *dhps* on chromosome 8 [35], [36], [37]. Primer sequences and their PCR parameters have been previously described [33], [38]. Close marker haplotypes around each gene were defined using codes previously developed for Peru [27], [29]. In addition, we examined 11 putatively neutral microsatellite loci including TA1, chromosome 6; poly α, ch. 4; PfPK2, Ch. 12; TA109, ch. 6; and 2490, ch. 10) [22], [39], as well as four markers on ch. 2, C2M33, C2M34, C2M29, C2M27 and three on ch. 3, C3M40, C3M69, and C3M39 [33]. We plan to share our data at the Worldwide Antimalarial Resistance Network website (www.wwarn.org) later in 2013.

### Statistical analysis

All tests in this study used an α of 5. For analyses of population structure, network diagrams, F_ST_ values, and bottleneck tests, we used seven neutral markers (TA1, chromosome 6; poly α, ch. 4; PfPK2, Ch. 12; TA109, ch. 6; and 2490, ch. 10), as well as four markers from each chromosome carrying one of four drug resistance genes, *dhfr*, *dhps*, *pfcrt*, and *pfmdr1* (Ch. 4, 347.1 kb; Ch. 5, −305 kb; Ch. 7, −257 kb; and Ch. 8, −196.6) selected to be as far from the genes as possible and exhibited H_e_ at or above our neutral estimates. These markers were used in a previous publication for the same purpose [40]. An analysis of molecular variance (AMOVA) was used to partition variation between all states, as well as between the coast vs. the interior. These tests were separately conduced for isolates collected during the 1980s and the 1990s. We also compared isolates collected in Pará during the 1980s and the 1990s. These tests were conducting using Arlequin version 3.1 [41]. Significance of the fixation indices was determined using a non−parametric approach. F_ST_ was calculated among all populations. The significance of F statistics and genetic variance components were tested using 1,000 permutations [41]. Isolation by distance was tested by regressing pairwise F_ST_ on pairwise geographic distances among populations [42] and significance determined with Mantel's tests (1,000 permutations) also using Arlequin [41].

We tested for bottlenecks using Bottleneck (www.ensam.inra.fr), which assumes that utilized markers are neutral and not in LD, and that populations lack substructure, migration, and hybrids. When a population is at mutation drift equilibrium (MDE), each microsatellite should have an equal probability of having an observed H_e_ deficit or excess in comparison to the expected H_e_ based on the number of alleles. After a bottleneck, there will be a reduction in the number of alleles and H_e_ at polymorphic loci. However, allelic diversity decreases at a faster rate than H_e_ during a bottleneck. Therefore, a bottleneck is indicated if a significant number of loci have a H_e_ excess compared to that expected if the population was in mutation-drift equilibrium. Conversely, if there is H_e_ deficit, the population will also no longer be in MDE and a rapid population expansion is indicated. To test whether our populations were in MDE, we used a sign test, which assumes a null hypothesis of MDE, but has low statistical power. To test for H_e_ deficits and excesses, we used a Wilcoxon sign-rank test [43]. We used a two-phased model of mutation for all tests [43].

### Network Analysis

We created median-joining network diagrams using Network v. 4.516 (fluxus-engineering.com) [44]. Like the bottleneck tests, we used the same 11 microsatellites.

#### Heterozygozity curves

for microsatellites around each gene and its alleles were calculated based on all sites at two time points: the mid-1980s and the late 1990s. It was decided not to divide the data by site based on the lack of diversity and relatively low F_ST_ values.

## Results

### *dhfr* and *dhps* genotypes

**Figure 2:**
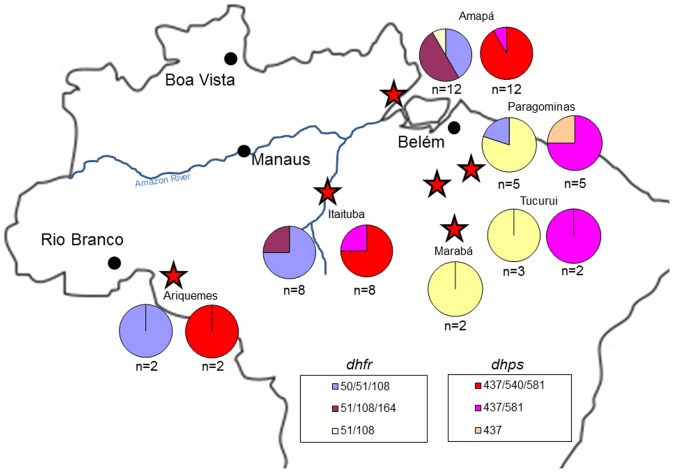
Geographic distribution of *dhfr* and *dhps* genotypes, 1980s. This figure shows the sites examined, noted with red stars, during the 1980s for *dhfr* and *dhps* genotypes. For each gene and site, there are two pie charts. The color coding for the alleles appears in the bottom of the map. Note that alleles with fewer mutations appear in the eastern portion of the country, while the 51I/108N/164L allele appears at a central and northeastern site.

**Figure 3:**
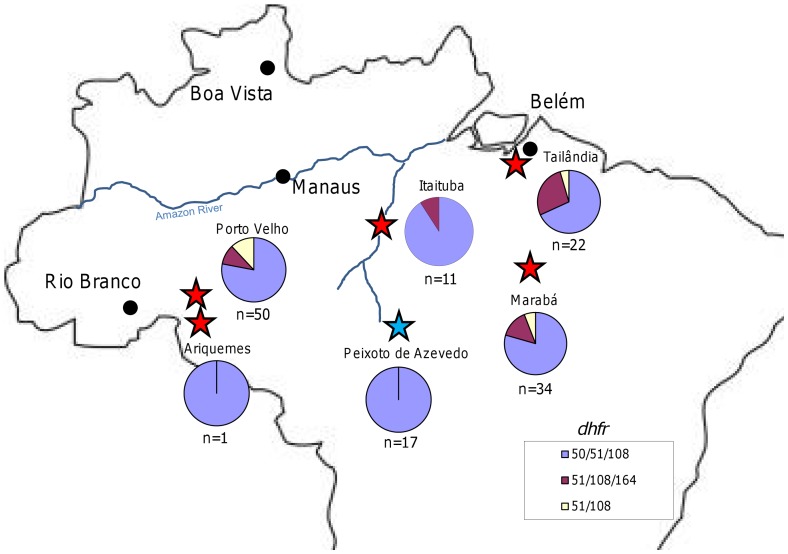
Geographic distribution of *dhfr* and *dhps* genotypes, 1990s. This figure shows the sites examined during the 1990s, noted with red stars, for *dhfr*. All sites were fixed for the 437G/540E/581G dhps allele, with the exception of one isolate in Itaituba carrying 437G/581G, and therefore these pie charts were omitted The color coding for *dhfr* appears in the bottom of the map. The data provided for Peixoto de Azevedo, noted by the blue star came from the work of others [45].

Parasites collected at all time points and locations in Brazil carried one of three *dhfr* alleles (51I/108N, 50R/51I/108N, or 51I/108N/164L). The temporal and geographic breakdown of these alleles is given in Table 2 and Figures 2 and 3. The double mutant *dhfr* allele occurred on two different close marker haplotypes as did each of the two triple mutant genotypes. However, almost all 51I/108N/164L occurred on the A1 background, while almost all of the 50R/51I/108N occurred on the D1 background. Taken together, this suggests that double mutants arose twice and that the triple mutants arose from different double mutant alleles.

**Table 2:** Temporal and geographic distribution of *dhfr* alleles in Brazil.

Site	Year	n =	Genotype-Haplotype
Amapá	1983	1	51I/108N-A1
Paragominas, Para	1983	1	51I/108N-A1
-	1983	1	51I/108N-D1
-	1984	1	51I/108N-D1
-	1985	1	51I/108N-D1
Tucuruí, Para	1983	1	51I/108N-D1
-	1984	2	51I/108N-D1
Marabá, Para	1984	1	51I/108N-A1
-	1984	1	51I/108N-D1
-	1999	1	51I/108N-D1
Porto Velho, Rondônia	1996	2	51I/108N-D1
-	1998	1	51I/108N-A1
-	1998	2	51I/108N-D1

Site	Year	n =	Genotype-Haplotype
Amapá	1984	1	51I/108N/164L-A1
-	1985	4	51I/108N/164L-A1
-	1986	1	51I/108N/164L-A1
Itaituba, Para	1985	1	51I/108N/164L-A1
-	1986	1	51I/108N/164L-A1
-	1999	1	51I/108N/164L-A1
Porto Velho, Rondônia	1996	3	51I/108N/164L-A1
-	1998	2	51I/108N/164L-D1
-	1998	1	51I/108N/164L-A1
Tailândia, Para	1998	5	51I/108N/164L-A1
-	1998	2	51I/108N/164L-D1
Marabá, Para	1999	5	51I/108N/164L-A1

Site	Year	n =	Genotype-Haplotype
Itaituba, Pará	1983	1	50R/51I/108N-A1
-	1983	1	50R/51I/108N-D1
-	1984	1	50R/51I/108N-D1
-	1985	2	50R/51I/108N-D1
-	1986	1	50R/51I/108N-D1
-	1999	10	50R/51I/108N-D1
Ariquemes, Rondônia	1984	1	50R/51I/108N-D1
-	1988	1	50R/51I/108N-D1
-	1996	1	50R/51I/108N-D1
Paragominas, Para	1984	1	50R/51I/108N-D1
Amapá	1985	5	50R/51I/108N-D1
Porto Velho, Rondônia	1996	12	50R/51I/108N-D1
-	1998	16	50R/51I/108N-D1
-	1999	11	50R/51I/108N-D1
Marabá, Para	1998	1	50R/51I/108N-A1
-	1998	17	50R/51I/108N-D1
-	1999	1	50R/51I/108N-A1
-	1999	8	50R/51I/108N-D1
Tailândia, Para	1998	1	50R/51I/108N-A1
-	1998	14	50R/51I/108N-D1

This table, broken into three pieces for readability, describes the genotypes and haplotypes of *dhfr* seen over time at different sites in Brazil.

**Figure 4:**
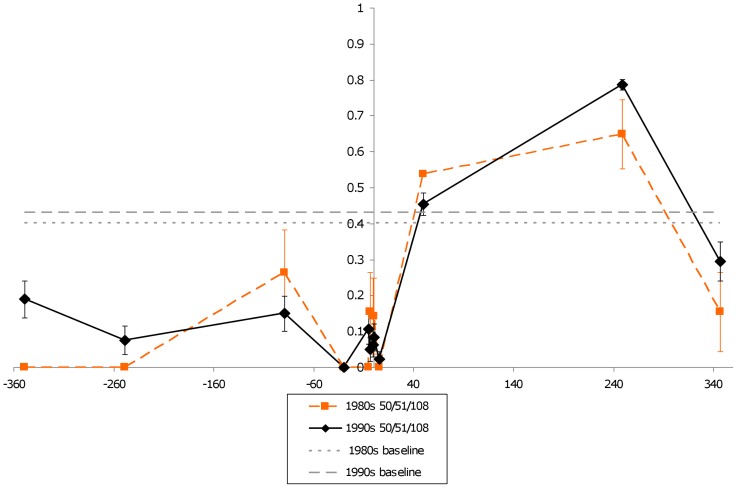
*dhfr* triple mutant H_e_ changes over time (50R/108N). The figure shows variation in H_e_ surrounding *dhfr* for parasites carrying a 51I/108N double mutant during the 1980s (n = 10) and the 1990s (n = 8). The two flat, dashed lines represent two different estimates of neutral H_e_ in Brazil.

**Figure 5:**
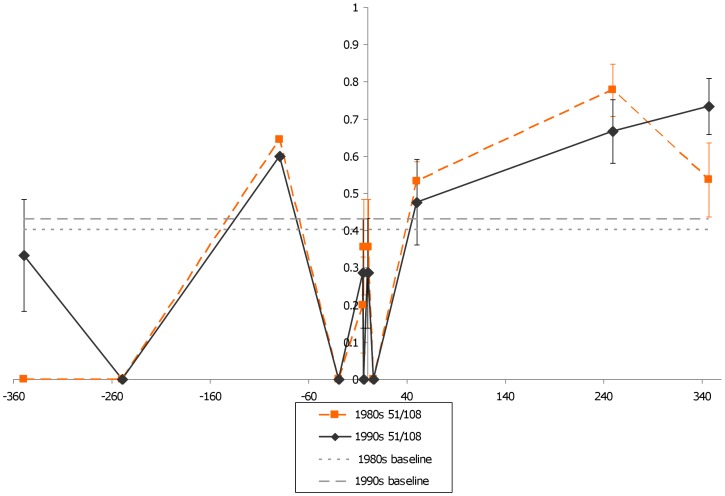
*dhfr* triple mutant H_e_ changes over time (50R/51I/108N). The figure shows variation in H_e_ surrounding *dhfr* for parasites carrying a 50R/51I/108N mutants during the 1980s (n = 14) and 1990s (n = 92). Note that H_e_ around the triple mutants seems similar between the two decades. The two flat, dashed lines represent two different estimates of neutral H_e_ in Brazil.

**Figure 6:**
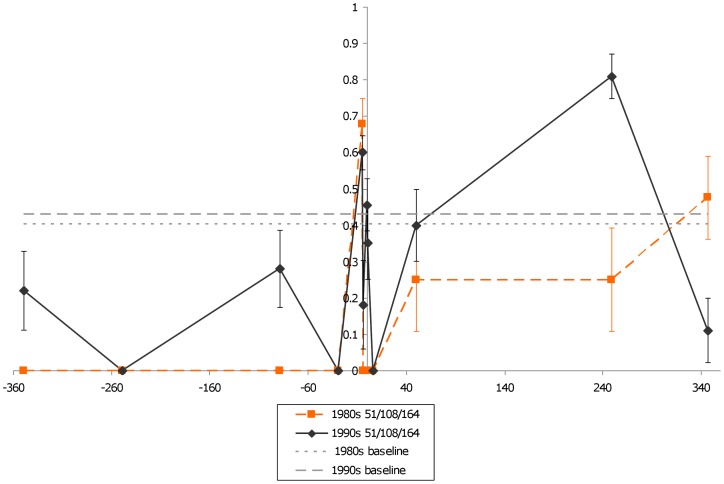
*dhfr* triple mutant H_e_ changes over time (51I/108N/164L). The figure shows variation in H_e_ surrounding *dhfr* for parasites carrying the 51I/108N/164L mutant during the 1980s (n = 8) and 1990s (n = 19). H_e_ surrounding this allele appears to have increased between the two periods. The two flat, dashed lines represent two different estimates of neutral H_e_ in Brazil.

**Figure 7:**
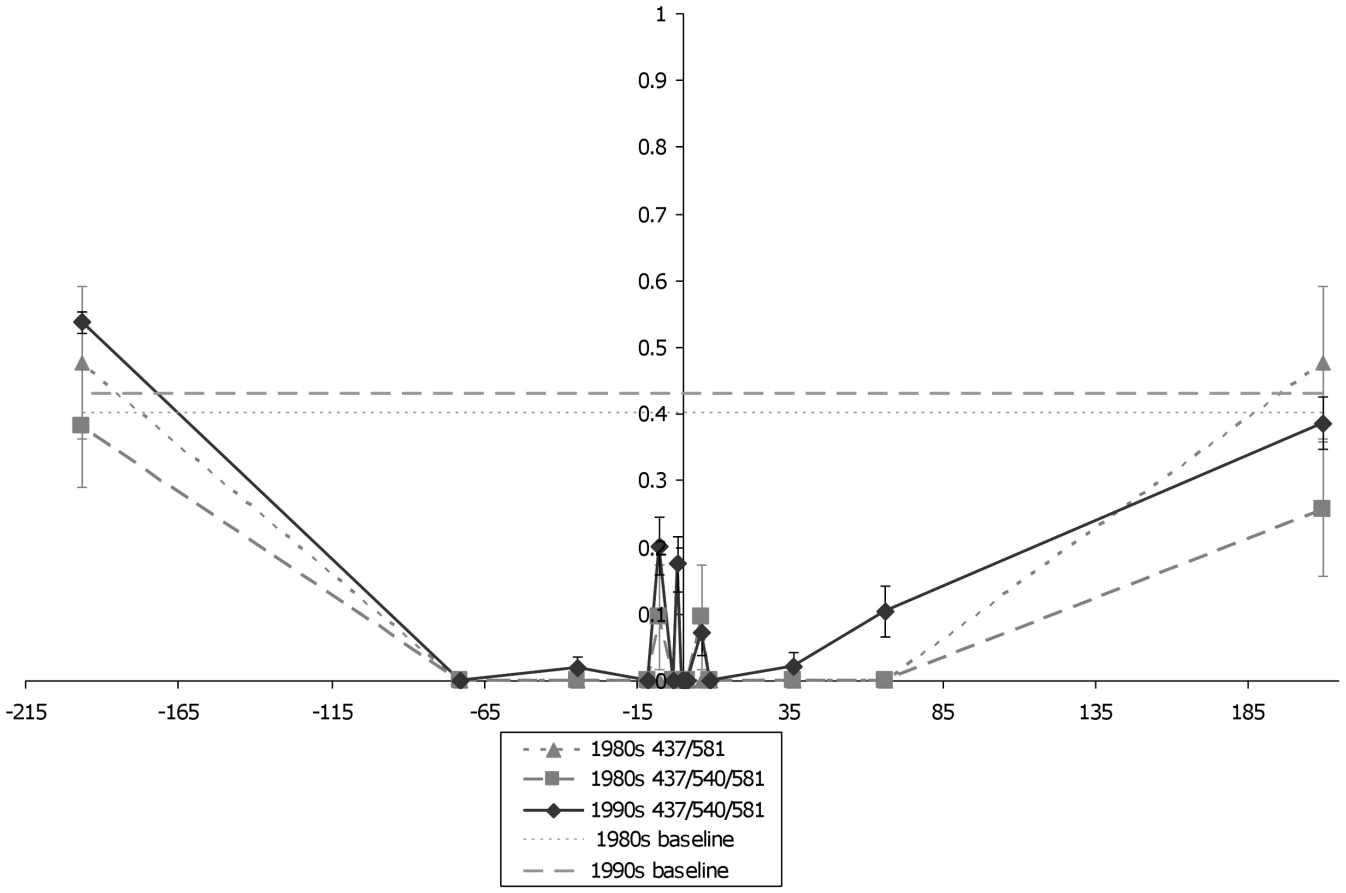
H_e_ around *dhps* from multiple periods across Brazil. The figure shows variation in H_e_ surrounding *dhps* during the 1980s and 1990s. It appears that H_e_ has increased between the 1980s (n = 21) and 1990s (n = 126) for the triple mutant. The double mutant (n = 7) seems to have a similar shape as the triple mutants. The two flat, dashed lines represent two different estimates of neutral H_e_ in Brazil.

Unlike earlier work, which only reported that the 51I/108N/164L *dhfr* allele in Brazil on the border with Bolivia and Peru [28], our data showed it was found at numerous sites throughout the country, including the eastern states of Pará and Amapa in the 1980s. This finding indicates that 51I/108N/164L allele may have been widespread as early as the 1980s in Brazil. Changes in H_e_ between the 1980s and 1990s for each gene are given in Figures 4–7. While the 50R/51I/108N genotype has maintained a similar low H_e_ curve during the 1980s and the 1990s, the 51I/108N/164L allele showed an increase in surrounding H_e_ during the 1990s indicating recombination had occurred. In comparison to these triple mutant alleles, the double mutant 51I/108N allele had a narrower valley of reduced H_e._

Parasites collected at all-time points and locations in Brazil carried the *dhps* 437G/540E/581G (156/166 of the samples sequenced) with the following exceptions. A sample collected during 1983 in Itaituba, Pará carried 437G/581G, as did another sample in 1999. One sample from 1983 and another from 1984 carried this genotype in Tucuruí, Pará. This genotype was also found in two samples collected in 1983, and another two collected in 1984 and 1985 in Paragominas, Pará. In 1983, Paragominas had one isolate that carried the single mutant 437G genotype. Information regarding which *dhfr* alleles these isolates carried are given in Table 3. Almost all samples collected in Brazil carried the same close *dhps* microsatellite haplotype, which we previously identified as A2 [27], [29]. Depressed microsatellite H_e_ for a 100 kb region around *dhps* suggest this allele had undergone strong selection (Fig. 7).

**Table 3:** Double mutant *dhfr* 51I/108N.

Site	Year	n =	*dhfr*	*Dhps*
Amapá	1983	1	51I/108N-D1	437/581-A2
Paragominas, Para	1983	1	51I/108N-A1	437-A2
	1983	1	51I/108N-D1	437/581-A2
-	1984	1	51I/108N-D1	437/581-A2
-	1985	1	51I/108N-D1	437/581-A2
Tucuri, Para	1983	1	51I/108N-D1	437/581-A2
-	1984	1	51I/108N-D1	437/581-A2
Marabá, Para	1999	1	51I/108N-D1	437/540/581-A2
Marabá, Para	1999	1	51I/108N-D1	437/540/581-A2
Porto Velho, Rondônia	1996	1	51I/108N-D1	437/540/581-A2
	1998	2	51I/108N-D1	437/540/581-A2

This table describes the SP resistant lineages seen in Brazil before the general fixation of triple mutants at various sites. ‘n  = ‘ denotes the number of isolates with this *dhfr*/*dhps* profile.

### Network Diagrams

**Figure 8:**
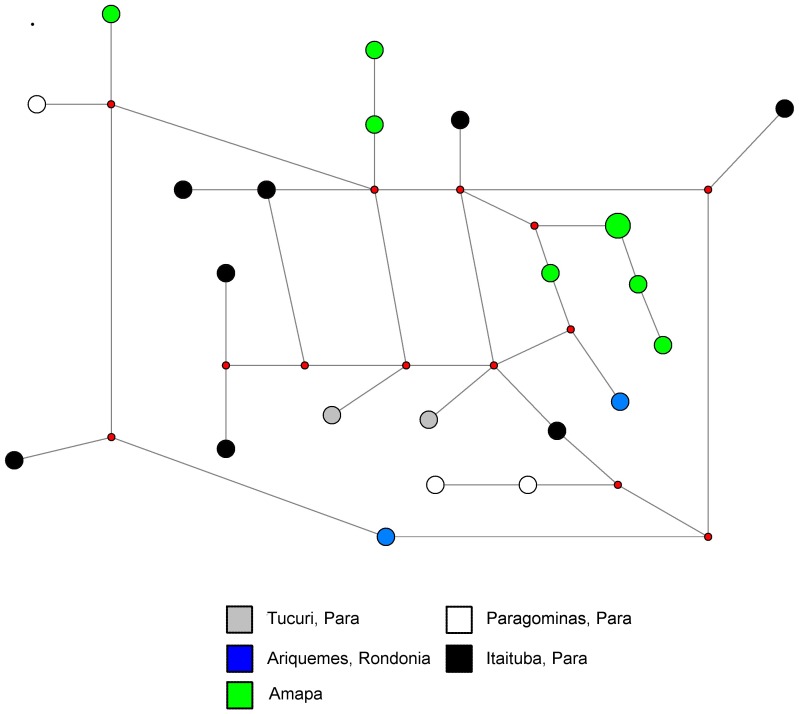
Network diagram of Brazilian data from the 1980s. In this median joining network diagram, we have included all samples collected in Brazil prior to 1996. Large colored circles come from the sites listed in the key and are proportional to the number of isolates with that microsatellite profile. The small red circles represent hypothetical nodes that link haplotypes seen among our samples.

**Figure 9:**
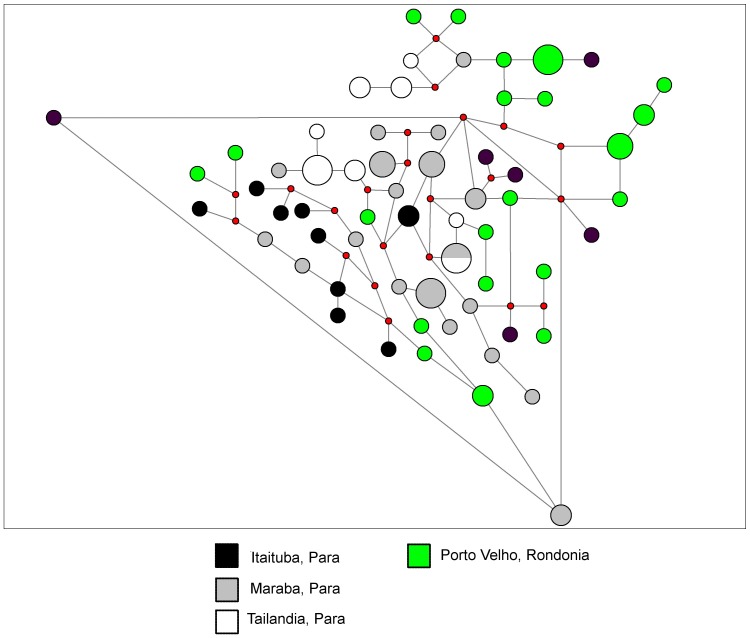
Network diagram of Brazilian data from the late 1990s. In this median joining network diagram, we have included all data that falls between 1996 and 1999. Large colored circles come from the sites listed in the key and are proportional to the number of isolates with that microsatellite profile. The small red circles represent hypothetical nodes that link haplotypes seen among our samples.

We report two network diagrams based on microsatellite neutral marker profiles. The network diagram in Figure 8 only includes data collected during the 1980s and is meant to represent historical population structure. It illustrates that none of the sites in Amapá, Pará, or Rondônia has a single group of parasite neutral haplotypes (though Tucuri, Pará haplotypes almost cluster). The network diagram in Figure 9 includes only data collected in 1996 or later. There is no strong evidence for clustering of neutral haplotypes, though there is one cluster at the top of the figure which includes all four sites. These data suggest that there is internal migration between sites causing admixture of parasite lineages.

### F_ST_ Analysis and AMOVA

This analysis was confined to only contemporaneous samples collected between 1996 and 1999 to control for shifts in parasite populations over time. Comparisons of sites during the 1980s are not reported due to limited sampling. The results of the F_ST_ analysis were generally significant with the exception of one comparison (Table 4). The sites that appeared most similar were Rondônia vs. Marabá, Pará (F_ST_  = 0.07) and Rondônia vs. Itatiuba, Pará (F_ST_ = 0.09). Interestingly, Tailândia, Pará did not share a similar low F_ST_ with Rondônia or the other sites in Pará.

**Table 4:** Pairwise F_ST_ of different sites in Brazil.

	Itaituba, Para	Marabá, Para	Moju, Para	Rondônia
Itaituba, Para				
Marabá, Para	0.04(p-value = = 0.11)			
Tailândia, Para	0.27(p-value = = 0.00)	0.21(p-value = = 0.00)		
Rondônia	0.09(p-value = = 0.00)	0.07(p-value = = 0.00)	0.30(p-value = = 0.00)	

This table shows pairwise F_ST_ of different sites using samples collected during 1996 to 2003. Rondônia consists of Porto Velho isolates, with the exception of one isolate from Ariquemes.

For AMOVAs conducted on data from the 1980s, grouping our data by state (Among groups: 6.5, p-value = 0.34; Among populations among groups: 9.5, p-value = 0.10; Within Populations: 84.0, p-value = 0.00) or coast vs. interior (Among groups: 6.7, p-value = 0.26; Among populations among groups: 9.6, p-value = 0.10; Within Populations: 84.0, p-value = 0.00) suggested most variation occurred within populations. The interior grouping for the aforementioned test consisted of isolates from Ariquemes and Itaituba, with all other isolates considered coastal.

For AMOVAs conducted on data from the 1990s grouping our data by state (Among groups: -1.3, p-value = 0.60; Among populations among groups: 17.5, p-value = 0.00; Within Populations: 84.0, p-value = 0.00) or coast vs. interior (Among groups:-0.5, p-value = 0.0.50; Among populations among groups:17.0, p-value = 0.00; Within Populations: 83.5, p-value = 0.00) also suggested most variation occurred within populations.

Our results for the AMOVA comparison of Pará isolates between the 1980s and 1990s suggested that much of the variation found in the 1990s was shared with that found in the previous decade (Among groups: -7.1, p-value = 0.93;Among populations among groups: 17.5, p-value = 0.00; Within Populations: 90.0, p-value = 0.00).

### Bottleneck Analysis

There were only a few sites and times with sufficient samples to conduct bottleneck analysis. Pará gave indications of recent bottlenecks according to both tests. Rondônia appeared to be in mutation drift equilibrium according to the H_e_ excess test, though not by the allele frequency distribution test (Table 5). The small sample size may explain this discrepancy.

**Table 5:** Tests for bottlenecks.

Location	Period	WilcoxinH_e_ deficiency	WilcoxinH_e_ excess.	Allele FrequencyMode Shift
Marabá, Para	1998–99	1.00	0.01	Yes
Tailândia, Para	1998	0.99	0.02	Yes
Tailândia, Para	1999	1.00	0.01	Yes
Porto Velho, Rondônia	1996–99	0.84	0.18	Normal L dist.
Porto Velho, Rondônia	1996	0.88	0.14	Yes
Porto Velho, Rondônia	1998–99	0.90	0.12	Yes

Two sites in Pará gave indications of bottlenecks during the 1990s based on the H_e_ excess test and the test for an Allele Frequency mode shift. During the 1990s, Porto Velho, Rondônia appeared to be in mutation drift equilibrium according to the H_e_ excess test though it trended towards significance. In addition, Porto Velho had a significant allele frequency distribution test when the 1990s data was divided between 1996 and 1998–1999m which suggests there had been a bottleneck.

## Discussion

The hypothesis that Brazilian *P. falciparum* has undergone bottlenecks followed by migration between sites is supported by its limited genetic diversity and the rejection of isolation by distance in our study, as seen in earlier papers [20], [21]. Unlike Peru, where parasite population structure appeared to be defined by recently reproductively isolated clonal lineages [40], there appeared to be gene flow across the Brazilian Amazon basin. We were unable to divide Brazilian parasite populations by clonal lineages and pairwise F_ST_ were lower than those reported for Peru (Table 4). Our AMOVA results agreed with earlier studies that found most parasite diversity was found within sites in the Brazilian Amazon [22]. We argue that the population structure of modern Brazilian *Plasmodium falciparum* appears to be based on ongoing admixture and reassortment of parasite lineages.

While the data suggested there was substantial migration between sites in the Brazilian Amazon Basin, there was no obvious structure to that migration (Figures 8 and 9). Interestingly, the two sites in Pará had lower F_ST_ values in comparison to Rondônia than to each other. The low reported F_ST_ for the comparison of Porto Velho, Rondônia and Marabá, Pará is also supported by the findings of a previous paper [20]. Rondônia might have acted as a source population multiple times for different sites in Pará or multiple different parasites populations from Pará may have populated Rondônia. Furthermore, parasites in Pará had gone through statistically significant bottlenecks, but those in Rondônia had not. This finding is in conflict with an earlier study that reported a potential bottleneck in Rondônia [22]. However, our data from Rondônia had a skewed allele frequency distribution (indicative of a bottlenecked population) and almost significant H_e_ excess, which suggest that limited sample size may have influenced our results. Although our findings are generally consistent with several previous studies, there was one study which did not find evidence of recent bottlenecks in Pará, Rondônia, Acre, or Amapá.[20]. In comparison to this study, we used more microsatellites on more chromosomes, as well as a different mutation model in Bottleneck.

It has been previously argued that highly resistant alleles of *dhfr* and *dhps* spread from the southern Amazon based on the understanding that high levels of SP resistance had first developed on the border of Bolivia and Brazil [19], [28]. Furthermore, it was suggested that the triple mutants arose from a single origin, with the 51I/108N/164L allele spread in a north-northwest direction across the Amazon, while the 50R/51I/108N allele spread north-northeast [28]. Our results suggest that there may have been at least two origins of double mutant 51I/108N *dhfr* alleles, which then led to the two separate triple mutant alleles. Furthermore, isolates from the 1980s in Amapá and Itaituba, Pará carried the 51I/108N/164L *dhfr* allele, though none were found in Rondônia (Figure 2). It is possible that highly resistant *dhfr*/*dhps* alleles were able to spread from the Bolivian border to the eastern coast of Brazil between 1982 and 1984, though somewhat unlikely.

Therefore these results challenge the hypothesis that the 51I/108N/164L alleles arose on the border with Bolivia, rather suggesting that it may have developed in the center of the Brazilian Amazon. Supporting this alternate hypothesis, the first reports of RIII resistance in 1982 occurred between Manaus and Porto Velho, a fair distance from the Bolivian border [19]. Additionally, triple mutant alleles had apparently spread throughout much of the Brazilian Amazon by the 1980s, which suggests that triple mutant alleles arose earlier than 1982. Indeed, it has been suggested that the double mutant *dhfr* and *dhps* mutations could have existed in Brazil as early as the 1960s based on clinical reports [18], [28]. RII pyrimethamine resistance was first reported in Goiás in 1968, which is in the center-south of the country, and resistance to the sulfadoxine-pyrimethamine drug combination was first reported in Brazil in that locality in 1972 [17], [18], [19]. These various historical reports suggest triple mutant *dhfr* alleles could have originated somewhere in the center of the Brazilian Amazon.

Whatever the origin of triple mutant *dhfr* and *dhps* alleles, our results suggest they did not develop in coastal Para, as this state appears to have only had parasites carrying double mutant *dhfr* (51I/108N) and *dhps* (437/581) parasites before 1986. In fact, that year may be when highly resistant alleles began to spread into this region.

By the 1990s, the frequency and distribution of the different *dhfr* and *dhps* alleles appears to have changed. In particular, the *dhfr* 50R/51I/108N allele had spread throughout the country and appeared to dominate populations. While the *dhfr* 51I/108N and 51I/108N/164L alleles were still present, they appeared along the eastern and western peripheries of the country. Meanwhile, the triple mutant *dhps* allele had disseminated throughout the country. The presence of both *dhfr* triple mutants in Rondonia during the 1990s, and the fixation of the triple mutant *dhps* allele, is verified by isolates sequenced by others [28], [45]. The wide distribution of *dhfr* 50R/51I/108N and 51I/108N/164L alleles is also supported by the analysis of isolates collected in Mato Grosso during the 1990s, but the study had inconsistent reporting for other regions [45]. Some of the distribution of *dhfr* alleles in Brazil during the 1990s may be the product of accidental migration. However, the preponderance of the 50R/51I/108N *dhfr* alleles after the wide use of SP suggest that it may have had higher fitness in the presence of SP in comparison to the reduced frequency of 51I/108N/164L. Assuming the two alleles have undergone similar selective pressure, the higher overall variation around the 50R/51I/108N *dhfr* allele (Figure 5) in comparison to the 51I/108N/164L allele (Figure 6) during the 1980s suggests 51I/108N/164L may have had more time to sexually recombine, implying it is the older allele. Furthermore, it appears that the reduced H_e_ seen around the 51I/108N/164L allele during the 1980s reduced over the next decade (Figure 6) due to sexual recombination with parasites carrying 50R/51I/164L, based on a qualitative examination of microsatellite haplotypes.

Though we had historical samples collected during the 1980s, we would have required samples from earlier years to understand how SP resistance spread across the basin or to fully describe the complex internal migration of Brazilian parasites after the colonization efforts of the past decades. However, it is clear that SP resistance, at both the double and triple mutant stages, developed twice and potentially in different regions of the Brazilian Amazon. Brazil might have had clonal structure similar to Peru during the 1960–1970s due to the reduction in malaria cases and genetic drift in isolated populations, but any such clonal lineages may have been masked over time due to extensive admixture and reassortment. Our recent paper from Peru indicates that the evidence for a bottleneck can rapidly disappear after parasite migration [40]. On the other hand, Brazilian malaria control efforts never completely suppressed malaria and therefore populations may have maintained high enough effective population sizes to avoid clonal expansions. Indeed, our bottleneck tests rejected this possibility generally, though they suggest that coastal Pará populations went through bottlenecks. From the standpoint of public health policy, the Brazilian Amazon basin may have sufficient internal migration for drug resistance reported in any particular region to rapidly spread to other parts of basin under similar drug pressure.
